# Mutational profiling reveals PIK3CA mutations in gallbladder carcinoma

**DOI:** 10.1186/1471-2407-11-60

**Published:** 2011-02-08

**Authors:** Vikram Deshpande, Afamefuna Nduaguba, Stephanie M Zimmerman, Sarah M Kehoe, Laura E MacConaill, Gregory Y Lauwers, Cristina Ferrone, Nabeel Bardeesy, Andrew X Zhu, Aram F Hezel

**Affiliations:** 1 Massachusetts General Hospital, Harvard Medical School, 55 Fruit Street, Boston, Massachusetts 02114, USA; 2 Center for Cancer Genome Discovery, Harvard Medical School, Dana-Farber Cancer Institute, 44 Binney Street, Boston, Massachusetts 02115, USA; 3 James P. Wilmot Cancer Center, University of Rochester School of Medicine, 300 Elmwood Avenue, Rochester, NY 14642, USA

## Abstract

**Background:**

The genetics of advanced biliary tract cancers (BTC), which encompass intra- and extra-hepatic cholangiocarcinomas as well as gallbladder carcinomas, are heterogeneous and remain to be fully defined.

**Methods:**

To better characterize mutations in established known oncogenes and tumor suppressor genes we tested a mass spectrometric based platform to interrogate common cancer associated mutations across a panel of 77 formalin fixed paraffin embedded archived BTC cases.

**Results:**

Mutations among three genes, KRAS, NRAS and PIK3CA were confirmed in this cohort. Activating mutations in PIK3CA were identified exclusively in GBC (4/32, 12.5%). KRAS mutations were identified in 3 (13%) intra-hepatic cholangiocarcinomas and 1 (33%) perihillar cholangiocarcinoma but were not identified in gallbladder carcinomas and extra-hepatic cholangiocarcinoma.

**Conclusions:**

The presence of activating mutations in PIK3CA specifically in GBC has clinical implications in both the diagnosis of this cancer type, as well as the potential utility of targeted therapies such as PI3 kinase inhibitors.

## Background

Biliary tract cancer (BTC) includes a spectrum of invasive adenocarcinomas including cholangiocarcinomas arising from within the liver parenchyma, peri-hilar, or distal biliary tree, as well as carcinoma arising from the gallbladder (GBC). Regardless of the site of origin, these tumors display a remarkably similar histologic appearance, variable amount of gland formation, and an exuberant desmoplastic stromal reaction. These tumors share an anatomic origin in the biliary system; however, there are important differences in disease behavior, molecular profiles, and sensitivity to therapy. In general GBC tends to exhibit greater initial sensitivity to chemotherapy but confers a shorter overall survival compared with cholangiocarcinoma (CC)[1]. Historically, treatment for BTC has not taken into account the anatomic site of origin of the tumor or molecular profile and the mainstay of treatment is cytotoxic chemotherapy, as these tumors are commonly diagnosed at advanced stages when surgical resection is not an option.

While a spectrum of mutations in established oncogenes and tumor suppressors have been identified in BTC the real frequency of such mutations and the relationship of mutations with each other has been hard to define. KRAS, BRAF, EGFR, and PIK3CA mutations are found in subsets of both GBC and CC [2-11]. Mutations in the tumor suppressor genes CDKN2A, TP53 and SMAD4 have also been identified [12-16]. The relationship of these mutations to each other as well as the frequency of each mutation within subsets of BTC is not yet fully explored. Additionally, many established mutations identified in other cancer remain to be evaluated in BTC.

Increasingly, cancer genetics are being applied to assist in making therapeutic decisions in cancer treatment. HER2NEU gene amplification, EGFR, and KRAS mutation testing are all used routinely clinically to determine an individual's likelihood of benefit from treatment with specific targeted anti-cancer therapies [17-19]. Building on this paradigm emerging classes of drugs, such as BRAF inhibitors, are being tested at the earliest phases selectively in genetically pre-screened populations of patients who are believed to have the greatest potential for benefit [20]. Given that underlying tumor genetics may predict drug sensitivity- particularly in emerging classes of targeted anticancer agents- uncovering patterns of genetic change within BTC is critical to improving therapy as well as gaining insight into disease biology.

In order to better characterize genetics of these tumors we conducted genotyping across 77 formalin fixed paraffin embedded (FFPE) surgical specimens including GBC as well as both intra- and extra-hepatic CC using "OncoMap". OncoMap is a high-throughput mass-spectrometric based cancer gene mutation profiling platform incorporating a collection of OncoMap 3 core -460 assays interrogating known mutations in 33 cancer genes [21,22]. Using genomic profiling with OncoMap coupled with an analytical mutation-calling algorithm and orthogonal validation step, numerous mutations have been identified in genomic DNA from both frozen and FFPE tumor tissue with a high degree of specificity and sensitivity[21]. This approach was selected given the focus of this platform on selecting established mutations highlighting pathways with emerging therapies, as well as the previously observed genetic heterogeneity of BTC. The primary goal was to identify novel or "druggable" mutations in biliary carcinoma.

## Methods

### BTC Samples

Samples were identified with institutional review board (IRB) approval through a search of pathologic cases of the gallbladder and biliary tract - resected or biopsied- available from archived tissues at the Massachusetts General Hospital between 1998 and 2008. In total 33 gallbladder, 29 IHCC and perihilar, and 15 middle common bile duct and intra-pancreatic biliary carcinomas were included to capture the broadest range of BTC. Hematoxylin and eosin (H&E) stainings were evaluated to confirm the diagnosis and to identify samples with the greatest tumor cellularity (ideally > 50%). Tumors were classified based on anatomic origin within the biliary tree and placed in 3 groups- gallbladder, intra-hepatic and perihilar, and distal common bile duct (CBD) and intra-pancreatic. Histological evaluation by two expert pathologists was used to distinguish distal bile duct carcinomas from pancreatic ductal adenocarcinomas, with the distinction based primarily on the pattern of infiltration and the relationship of the tumor to the bile duct.

### Extraction of Tumor Genomic DNA

For FFPE samples, tissue from representative blocks with areas of >50% tumor cellularity were sectioned (five 10 micrometer sections) and serial H&E-stained slides were obtained from each block. Areas of > 50% tumor cellularity were dissected from slides using a scalpel and scraped into micro-centrifuge tubes. Diagnoses were confirmed by independent histopathological review. DNA was extracted from FFPE according to the manufacturer's directions. The quality of DNAs was evaluated by quantification (Picogreen) and PCR amplification of fragments of 100-200bp in length.

### Mass Spectrometric Genotyping

A two-step process was used as previously described [21], where candidate mutations identified using OncoMap technology were subsequently subject to a second round of homogenous Mass-Extend (hME) validation, using independent primers and probes and non-whole genome amplified DNA. In brief, primers and probes were pooled, and all assays were validated on the CEPH panel of human HapMap DNAs (Coriell Institute) as well as a panel of human cell lines with known mutational status, as described previously [21]. Genomic DNA from all tumor samples was quantified using Quant-iT™ PicoGreen^® ^dsDNA Assay Kit (Invitrogen, Carlsbad, California) and subjected to whole-genome amplification (WGA), as described previously[21]. After quantification and dilution of genome-amplified DNA, mass spectrometric genotyping using iPLEX chemistries was performed as described previously[21]. An automated mutation-calling algorithm was performed to identify candidate mutations and putative mutations were further filtered by a manual review. Candidate mutations identified were subsequently subject to a second round of hME validation, using independent primers and probes and a multi-base extension hME chemistry. Conditions for hME validation were as previously described [21]. All mutations identified in WGA material were confirmed, using a second independent assay, on unamplified DNA.

### Statistical Analysis

Fishers exact test was applied to determine the association of PIK3CA mutation with GBC.

## Results

### Identification of mutations in BTC

**Figure 1:**
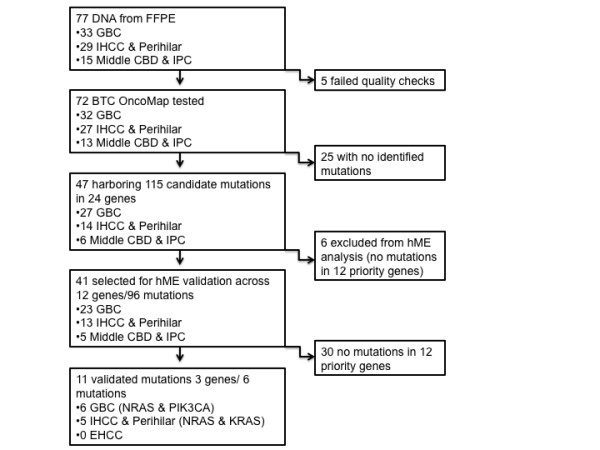
**Flow-chart of Identification of Mutations (Abbreviations: FFPE; formalin fixed paraffin embedded, GBC; gallbladder carcinoma, IHCC; intra-hepatic cholangiocarcinoma, CBD; common bile duct, IPC; intra-pancreatic cholangiocarcinoma)**.

Mutations in KRAS, NRAS, and PIK3CA were identified as outlined in Table 1 and 2. In summary the process leading to positive identification of mutations involved a two-step process was used as previously described [21], where candidate mutations identified using OncoMap technology (all mutations included in initial screen are listed in Additional file 1 Table S1) were subsequently subject to a second round of hME validation (for details see Figure 1). From among the 77 BTC the percentage of samples that were successfully genotyped indicating that intact DNA was recovered from paraffin blocks was 93.5%. Five samples failed quality control steps designed to evaluate overall quality of recovered DNA (quality control measures described in [22] and were not included in subsequent analysis (Figure 1). Of the 72 samples with good quality DNA, initial OncoMap screening indentified 115 candidate mutations across 24 genes in 65% (47/72) of samples. These were then ranked into two groups based on the mass spectrometric profiles and the likelihood that these represent true mutations referred to as conservative and aggressive "calls" which comprised 10% (12/115) and 90% (103/115) of possible mutations, respectively (outlined in Figure 1 and Additional file 1 Table S2).

**Table 1:** Mutation rates by tumor location

	Gallbladder Cancer (32)	Intra-hepatic CC (24)	Perihilar CC (3)	Middle CBD and Intrapancreatic CC (13)
KRAS	0 (0%)	3 (13%)	1 (33%)	0 (0%)
NRAS	2 (6.3%)	1 (4%)	0 (0%)	0 (0%)
PIK3CA	4 (12.5%)	0 (0%)	0 (0%)	0 (0%)

**Table 2:** Mutations in BTC

Gene	Mutation	Source
KRAS	G12A	Perihilar
KRAS	G12C	Intra-hepatic
KRAS	G12V	Intra-hepatic
KRAS	G12V	Intra-hepatic
NRAS	G12D	Gallbladder
NRAS	G12D	Gallbladder
NRAS	Q61R	Intra-hepatic
PIK3CA	E545K	Gallbladder
PIK3CA	E545K	Gallbladder
PIK3CA	E542K	Gallbladder
PIK3CA	E542K	Gallbladder

Candidate mutations across 12 genes (ABL1, APC, BRAF, EGFR, FGFR3, FLT3, KIT, KRAS, NRAS, PDGFRA, PIK3CA, MYC,) were then evaluated by an hME approach on non WGA DNA using independent primers and probes (for details on specific mutations screened see Additional file 1 Table S3). All samples that included a candidate mutation of interest were included in the hME analysis. Candidate gene mutations included in validation assays were chosen based on a high prevalence of mutations, potential for targeting with therapeutics, potential clinical utility, or novelty. Activating mutations in KRAS, NRAS, and PIK3CA were confirmed. Among these activating PIK3CA mutations were unique to gallbladder tumors.

### Mutations in gallbladder carcinoma and clinicopathologic correlates

33 gallbladder carcinomas were analyzed (Table 3). The mean age of this cohort was 66 years, and included 25 females and 8 males. Additionally, two gallbladders with high-grade dysplasia were also evaluated. The majority (n = 27) of these cases were adenocarcinomas. In addition, we analyzed 3 adenosquamous carcinomas, and one case each with the following histologic features: squamous cell carcinoma, colloid carcinoma, and undifferentiated carcinomas. PI3K mutations were specific to carcinomas of the gallbladder and were identified in 4 (12.5%) gallbladder carcinomas, (p = 0.013 vs. CC). Of the 4 cases with mutations, 3 were identified in adenocarcinomas, and 1 in an adenosquamous carcinoma. There are no appreciable differences in T stage or patient characteristics between the cohort that showed PI3K mutations and the group of cases that did not (Table 3). Neither of the two gallbladders with high-grade dysplasia showed PI3K mutations.

**Table 3:** Clinical characteristics of profiled GBC

Mutation	Gender	Smoking	Age	T	N	M	Stage	Histology	Differentiation
PIK3CA E545K	F	yes	75	1	2	0	1	adenocarcinoma	Moderate
PIK3CA E542K	F	no	83	2	2	0	1	adenocarcinoma	Poor
PIK3CA E542K	M	yes	82	3	1	0	2	adenocarcinoma	Moderate
PIK3CA E545K	F	unknown	53	3	0	0	2	adenosquamous carcinoma	Poor
NRAS G12D	F	no	56	2	1	1	4	adenocarcinoma	Poor
NRAS G12D	F	yes	76	2	0	1	4	adenocarcinoma	Moderate
	F	no	68	3	2	0	2	adenosquamous carcinoma	Poor
	F	no	62	1	2	0	1	adenocarcinoma	Moderate
	F	no	62	3	1	0	2	adenosquamous carcinoma	Poor
	F	unknown	79	4	2	0	3	adenocarcinoma	Moderate
	F	yes	72	3	2	0	2	adenocarcinoma	Unknown
	F	no	57	3	2	0	2	adenocarcinoma	Poor
	M	yes	55	2	1	0	2	adenocarcinoma	Poor
	F	no	87	3	1	0	2	adenocarcinoma	Moderate
	F	no	63	1	0	0	1	adenocarcinoma	Moderate
	M	unknown	54	3	2	0	2	adenocarcinoma	Poor
	M	no	48	3	0	0	2	adenocarcinoma	Poor
	F	yes	52	3	2	1	4	adenocarcinoma	Poor
	F	no	60	3	0	1	4	adenocarcinoma	Poor
	F	yes	76	2	2	0	1	adenocarcinoma	Poor
	F	yes	75	1	2	0	1	adenocarcinoma	Moderate
	F	no	66	4	2	0	3	Undifferentiated carcinoma	Poor
	F	yes	57	4	2	1	4	adenocarcinoma	Unknown
	F	no	72	2	0	0	1	adenocarcinoma	Moderate
	M	yes	81	3	0	1	4	adenocarcinoma	Poor
	F	no	41	2	0	0	1	adenocarcinoma	Unknown
	M	no	68	2	0	0	1	adenocarcinoma	Poor
	M	yes	75	3	0	0	2	adenosquamous carcinoma	Moderate
	F	yes	70	2	2	0	1	adenocarcinoma	Poor
	F	no	74	3	2	0	2	adenocarcinoma	Moderate
	M	no	63	3	2	0	2	adenocarcinoma	Poor
	F	yes	58	2	0	0	1	adenocarcinoma	Poor
	F	no	67	3	0	1	4	adenocarcinoma	Unknown

### Mutations in intrahepatic and perihilar cholangiocarcinomas

The cohort included 24 intra-hepatic cholangiocarcinomas in which 3 (13%) KRAS mutations and 1 NRAS (4%) mutation were identified (Table 1). We did not identify mutations in the single mixed hepatocellular-cholangiocarcinoma evaluated.

### Mutations in perihilar cholangiocarcinomas

One of the three peri-hilar cholangiocarcinomas harbored a mutation in KRAS.

### Mutations in mid bile duct carcinomas and intrapancreatic cholangiocarcinomas

This group included 6 adenocarcinomas arising from the middle third of the bile duct and 9 adenocarcinomas arising from the intrapancreatic portion of the bile duct. None of these cases showed mutation among KRAS, NRAS or BRAF.

## Discussion

Mutations in KRAS, NRAS and PIK3CA, all of which have previously been identified in biliary tract carcinomas (for summary of mutations in BTC see Table 4), were identified in this mutational screen (Reviewed in [23]) [3,6,24,25]. Furthermore, PIK3CA mutations appear to be confined to gallbladder carcinomas among this cohort. Reiner and co-workers have previously identified PIKCA mutations in one of 11 intra-hepatic cholangiocarcinoma and one of 23 GBC previously[11]. However, an association of PIK3CA mutations with GBC was not appreciated.

**Table 4:** Mutational Spectrum of Oncogenes and Tumor Suppressor Genes

Gene	GBC (%)	EHCC (%)	IHCC (%)	Method	**Refs**.
**CTNNB1/** **β-CATENIN**	5			SEQ	[33]
	9	0		SEQ	[34]
	0			SEQ	[35]

**KRAS**	38			PCR-SSCP	[5]
	20			PCR-RFLP	[16]
	19			PCR-RFLP	[32]
	3	15		SEQ	[3]
		10		PCR-SSCP	[15]
			54	SEQ	[6]
			45	SEQ	[2]
			48	SEQ	[36]
	0	0	13	OncoMap	

**BRAF**	33			SEQ	[7]
			22	SEQ	[2]
	0	0	0	SEQ & GLCR	[8]

**EGFR**	9	18	20	SEQ	[9]
		6		SEQ	[10]
	12	5	10	IHC and FISH	[37]

**PIK3CA**	4	0	9	SEQ	[11]
	13	0	0	OncoMap	

**ERBB2/** **HER-2**	16	5	0	IHC and FISH	[37]

**P16INK4A**	31			SSCP	[16]
	62	55		numerous	[13]
			88	numerous	[6]

**TP53**	36			SSCP	[16]
		33		PCR-SSCP	[15]
			37	SEQ	[14]

**SMAD4**		16		PCR-SSCP	[12]
		55	13	IHC	[38]

**STK11/LKB1**		6		SEQ	[39]

Abbreviations: GBC; gallbladder carcinoma, IHCC; intra-hepatic cholangiocarcinoma, EHCC; extra-hepatic cholangiocarcinoma, SEQ; sequencing, PCR-SSCP; polymerase chain reaction single-strand confirmation polymorphism, RFLP; restriction fragment length polymorphism, GLCR; gap ligase chain reaction.

Somatic mutations of the PIK3CA gene, which encodes the p110alpha catalytic subunit of phosphatidylinositol 3-kinase (PI3K), are found across a range of cancers with the highest rates of mutation observed in breast, colon, endometrial, bladder and hepatocellular cancers (COSMIC data base). The majority of mutations cluster at hotspots within exons 9 and 20, which encode the helical and kinase domains of p110alpha and lead to activation of downstream pro-growth and survival pathways [26]. Importantly mutations in these domains render cancers sensitive to PI3K specific inhibitors pointing towards a role for this emerging class of drugs in cancers harboring these mutations [27]. The PIK3CA mutations identified in this study, E542K and E545K, are both located in exon 9.

Activation of the PI3K pathway can be achieved through a number of molecular mechanisms, including loss of the PTEN tumor suppressor gene, mutation in EGFR, and amplifications of ERBB2 (HER2NEU), as well as through mutation of PIK3CA, as described above [26,28,29]. ERBB2 over-expression and gene amplification is found in ~ 15% of GBC[30]. Additional evidence implicating the importance of this pathway in BTC comes from mouse models. A transgenic mutant with constitutive expression of the ErbB2 in the gallbladder epithelium develops GBC with a 100% penetrance [30] and somatic mutation of *Pten *leads to biliary hyperplasia and intraheaptic cholangiocarcinoma [31]. Taken together this data points towards deregulation of PI3K signaling as a key event in the molecular pathogenesis of some subsets of BTC.

KRAS gene mutations are identified in this series. The majority of mutations are identified in intra-hepatic cholangiocarcinomas (13%) and peri-hilar adenocarcinomas (33%). KRAS mutations were not identified in gallbladder carcinomas and extra-hepatic cholangiocarcinomas. In general, reported rates of KRAS mutation are somewhat lower in these sites than intra-hepatic cancers (3-20%)[3,16,32] with a notable exception being neoplasms that arise in the setting of an anomalous union of the pancreatic and biliary ducts, which have higher rates of KRAS mutation[5]. None of the cases in this series had evidence of an anomalous anatomy of the bile ducts. While our results from the gallbladder are similar to that reported in literature, the data on extra-hepatic neoplasm differs from prior reports that suggest 10-15% of extra-pancreatic cholangiocarcinomas harbor mutations in the KRAS gene[3,15]. In this series, KRAS mutations were not identified in any of the adenocarcinomas arising from the mid-portion of the bile duct or the intra-pancreatic portion of the bile duct. It is possible that the KRAS mutations identified in extra-hepatic bile duct carcinomas in prior studies may represent cases of peri-biliary pancreatic ductal adenocarcinoma. Histology remains the gold standard for distinguishing distal bile duct carcinomas from pancreatic ductal adenocarcinomas, and this distinction is based primarily on the pattern of infiltration and the relationship of the tumor to the bile duct. Nonetheless, there is significant overlap in the histologic appearance of these two neoplasms. We did not detect any KRAS mutation among the 13 distal extra-hepatic and intrapancreatic biliary carcinoma. Analysis of the absence of KRAS mutations in extra-hepatic and intrapancreatic biliary carcinoma in comparison to the 4 mutations identified intrahepatic and perihilar cholangiocarcinomas shows this relationship to be insignificant, p = 0.28 (Fishers exact test). It is possible that the absence of KRAS mutations in extra-hepatic cancers in our screen was a chance event. It is also, however, possible that intra-hepatic and perihilar cholangiocaricinomas may differ genetically from more distal bile duct carcinomas.

While this study identified known mutations and points towards an association between PIK3CA mutations and GBC, the rates of validated mutations in genes such as KRAS, particularly in the intra-hepatic cholangiocarcinomas, were lower than expected given the findings of previous studies[2-6]. Additionally, other previously identified mutations in genes such as BRAF and EGFR were not identified here[2,10]. Potential reasons for this could include a diminished sensitivity of the assay among our sample set. This could be due to dilution of tumor-derived nucleotides by tumor associated desmoplasia and stroma- a common feature of BTC. By selecting tumor specimens with ~50% or higher cellularity we had attempted to diminish the impact of this. Alternatively, these mutations may be present at only a low incidence or not at all. Among the three previously published studies BRAF mutations are identified in ~20% of cases in two European BTC collections including both GBC and intra-hepatic CC [2,7]. No BRAF mutations were found in an American cohort [8]. This is echoed in the above findings. In spite of the extensive mutational analysis (Additional file 1 Table S1 and Additional file 1 Table S3), we did not identify any additional new mutations in biliary tract carcinomas.

## Conclusions

The molecular characterization of subgroups of tumors has become critical to development of new classes of anti-cancer agents. Inhibitors of the PI3K pathways are presently in development including specific inhibitors of p110alpha that have demonstrated efficacy in engineered preclinical models of lung cancer harboring activating point mutations in PIK3CA[27]. These results, pointing towards an enrichment of activating PIK3CA mutations in GBC, suggest a potential group of patients with GBC who may also benefit from these agents. Further mutational profiling efforts across larger cohorts of BTC are needed to confirm these results and better define genetically distinct subsets of cancers.

## Competing interests

The authors declare that they have no competing interests.

## Authors' contributions

VD, NB, AZ, and AH conceived of the experimental plan and methodologies. Pathologic analysis and interpretation was provided by VD and GL. VD, AH, AN, SZ carried out experiments. Data was collected by VD, AF, SZ, CF. SK and LM conducted mutational screening, analysis, and validation. All authors were involved in writing the paper and had final approval of the submitted and published versions.

## Pre-publication history

The pre-publication history for this paper can be accessed here:

http://www.biomedcentral.com/1471-2407/11/60/prepub

## Supplementary Material

Additional file 1 **Supplementary Table 1-3**. Table S1: All mutations included in initial OncoMap screen. Table S2: Listing of mutations identified in initial OncoMap screening. 115 candidate mutations across 24 genes in 65% (47/72) of samples were identified. These were then ranked into two groups based on the mass spectrometric profiles and the likelihood that these represent true mutations referred to as conservative and aggressive "calls" which comprised 10% (12/115) and 90% (103/115) of possible mutations, respectively. Table S3: Candidate mutations across 12 genes (ABL1, APC, BRAF, EGFR, FGFR3, FLT3, KIT, KRAS, NRAS, PDGFRA, PIK3CA, MYC,) screened in hME validation assay on non WGA DNA using independent primers and probes. Click here for file
